# Adherence to management guidelines for growth faltering and anaemia in remote dwelling Australian Aboriginal infants and barriers to health service delivery

**DOI:** 10.1186/1472-6963-13-250

**Published:** 2013-07-03

**Authors:** Sarah J Bar-Zeev, Sue G Kruske, Lesley M Barclay, Naor Bar-Zeev, Sue V Kildea

**Affiliations:** 1 Centre for Rural Health, North Coast; Sydney School of Public Health, University of Sydney, New South Wales, Sydney 2480, Australia; 2 Queensland Centre for Mothers and Babies, School of Psychology, University of Queensland, Brisbane, Queensland 4072, Australia; 3 Centre for Rural Health; North Coast, University of Sydney, New South Wales 2480, Sydney, Australia; 4 Menzies School of Health Research, Charles Darwin University, Darwin, 0909, Sydney, Australia; 5 Midwifery Research Unit, Australian Catholic University and the Mater Medical Research Institute, Queensland 4010, Brisbane, Australia; 6 Malawi-Liverool-Wellcome Trust Clinical Research Programme, Institute of Infection and Global Health, University of Liverpool, Liverpool, Merseyside L69 3BX, UK

**Keywords:** Aboriginal, Adherence, Anaemia, Australia, Barriers, Growth faltering, Infant, Management guidelines, Primary health care, Remote, Quality of care

## Abstract

**Background:**

Remote dwelling Aboriginal infants from northern Australia have a high burden of disease and frequently use health services. Little is known about the quality of infant care provided by remote health services. This study describes the adherence to infant guidelines for anaemia and growth faltering by remote health staff and barriers to effective service delivery in remote settings.

**Methods:**

A mixed method study drew data from 24 semi-structured interviews with clinicians working in two remote communities in northern Australia and a retrospective cohort study of Aboriginal infants from these communities, born 2004–2006 (n = 398). Medical records from remote health centres were audited. The main outcome measures were the period prevalence of infants with anaemia and growth faltering and management of these conditions according to local guidelines. Qualitative data assessed clinicians’ perspectives on barriers to effective remote health service delivery.

**Results:**

Data from 398 health centre records were analysed. Sixty eight percent of infants were anaemic between six and twelve months of age and 42% had documented growth faltering by one year. Analysis of the growth data by the authors however found 86% of infants experienced growth faltering over 12 months. Clinical management and treatment completion was poor for both conditions. High staff turnover, fragmented models of care and staff poorly prepared for their role were barriers perceived by clinicians’ to impact upon the quality of service delivery.

**Conclusion:**

Among Aboriginal infants in northern Australia, malnutrition and anaemia are common and occur early. Diagnosis of growth faltering and clinicians’ adherence to management guidelines for both conditions was poor. Antiquated service delivery models, organisation of staff and rapid staff turnover contributed to poor quality of care. Service redesign, education and staff stability must be a priority to redress serious deficits in quality of care provided for these infants.

## Background

Health outcomes of Australian Aboriginal children are significantly worse than their non-Aboriginal counterparts [1]. These differences are manifest in two to three times higher rates of perinatal mortality, preterm birth and low birth weight [2]. The prevalence of anaemia and nutritional problems is much higher in Aboriginal children during their first years of life as is the overall burden of disease and hospitalisation rate [3-6]. Prevention of nutritional disorders during infancy is imperative as early growth and development form the foundation for health and learning throughout the rest of life [7]. Iron deficiency anaemia is the leading type of anaemia identified in remote dwelling Aboriginal children in the Northern Territory (NT) [8]. It is thought to result from low birth weight, chronic infection, delayed introduction and inadequate intake of iron rich foods and high rates of parasite and worm infestation which cause diarrhea, growth faltering and malabsorbtion [9-11]. Numerous studies have shown associations between iron deficiency anemia and delayed psychomotor development and behavioural problems in childhood [12-15].

Most Aboriginal people in Australia live in cities and regional areas; with one quarter residing in remote communities [16]. Aboriginal people living in these communities tend to have worse health outcomes than those in urban or larger rural areas [17].

Aboriginal infants in the Northern Territory frequently use remote primary health services in their first year of life, mostly for acute illness [18,19]. Growth and anaemia monitoring are also common reasons for primary health service use [19]. Despite the frequent use of services, data describing the quality of services are limited [20,21]. We therefore aimed to measure the quality of service delivery provided to Aboriginal infants in remote health centres (RHCs) against local guidelines. Growth faltering and anaemia were selected as quality indicators given their high prevalence in remote NT communities and the importance of their management from an early age for long term health [7]. This study also sought to identify barriers to effective health service delivery in these RHCs.

This paper reports on baseline data from the ‘1 + 1 = A Healthy Start to Life’ project. It used a participatory approach and a mixed method design to inform interventions led by health service staff to improve maternal infant care for remote dwelling families in northern Australia. The project was developed in response to concerns voiced by Aboriginal women, policy makers and clinicians about the quality of maternal and infant health services.

### Setting

Study sites were RHCs in two large (population 2200–2600) remote Aboriginal communities situated around 500 km from the major referrral hospital in Darwin. Darwin is a small capital city with health services out of proportion for its large catchment area. It located is in the Top End of the NT of Australia.

Within the remote communities English is typically the second or third language, unemployment common and family income among the lowest in the country [22].

Remote health centres are open during business hours with staff ‘on call’ for emergencies. Most health care is provided by registered nurses (RNs), midwives and Aboriginal Health Workers (AHWs) within the HCs. Aboriginal health workers (AHWs) are felt to ‘bridge to the cultural chasm’ dividing the Indigenous and non-Indigenous ideologies, thus acting as a cultural broker as well as primary health care worker [23]. Aboriginal Health Workers provide ‘clinical and primary health care for individuals, families and community groups. They deal with patients, clients and visitors to hospitals and health clinics and assist in arranging, coordinating and providing health care in Aboriginal and Torres Strait Islander community health clinics’ [24].

An onsite doctor sees patients on a referral basis. Outreach paediatricians and child health nurses from Darwin visit regularly. There are no in-patient beds in the RHCs so infants requiring medical evacuation are flown to the regional hospital.

The burden of disease and the use of RHCs by infants from these communities is very high, commencing from birth and continuing throughout the first year. Twenty one percent of all infants born 2004–2006 were pre-term and 18% were low birth weight. One third of infants were admitted to the regional hospital neonatal nursery, primarily for preterm birth, low birth weight and presumed sepsis.

Infants had a mean of 28 presentations to the RHCs per year, with half of all visits for new, acute problems. Remaining presentations were for reviews or routine health service provision such as growth monitoring and immunisation. By age one 59% of infants were admitted to hospital at least once, most commonly for respiratory infections (47%), gastroenteritis (27%) and failure to thrive (5%). The rate of hospitalisation per infant year was 1.1 (95% CI 0.9-1.2) [19].

Anaemia and growth faltering are major child public health problems in remote NT communities [25] and thus require population based approaches for their prevention and management. At the time of data collection the Growth Action and Assessment program (GAA) was the main health program for remote dwelling children under five years. It has since been superseded by the Healthy Under 5 Kids Program [26]. Growth Action and Assessment was implemened in the NT in the 1990’s to address poor nutrition - one of the leading causes of morbidity in remote Aboriginal children. It was designed to tackle growth and nutritional issues using surveillance, monitoring and treatment guidelines outlined in a local Standard Treatment Manual [27] used for common infant presentations in remote practice. These guidelines are designed to be used by all remote clinicians and standardise care.

There is a high turnover and on-going shortage of nursing and midwifery staff working in remote settings [28] and those with midwifery and child health qualifications have declined from 65% and 18%, respectively in 1995 to 29% and 11% in 2008 [29]. This is thought to result from modifications that have been made to post-graduate nurse education and the expanded choice of post-graduate courses on offer [29].

Ethics approval was obtained from the Human Research Ethics Committee of the Menzies School of Health Research, and remote community leaders. Written consent was obtained from interview participants.

## Methods

We used a mixed-methods approach [30] integrating a retrospective cohort study and interviews with clinicians to identify barriers to high quality remote health service delivery. Data was collected between January-August 2008.

### Retrospective cohort study

We undertook a retrospective cohort study of all Aboriginal infants from these communities, collecting data from birth to age one. Infants born 1 January 2004 to 31 December 2006 with gestation of at least 20 weeks or birth weight of at least 400 grams and born at the regional centre hospital, in hostel accommodation, in transit to hospital or in the remote community, were included. We constructed the study cohort through manual data linkage between community birth records and medical records at the two HCs and the regional hospital, identifying 424 eligible infants. Of these infants, 11 (2.6%) had no community or hospital record. The final cohort consisted of 413 infants; 398 of these infants had a remote health centre record available for review. There were 2 neonatal and 2 infant deaths in this study.

Data were collected by manually reviewing the infants’ medical record at the RHCs. We collected data in Table 1 and assessed it against guidelines [27] for the identification and management of infants with anaemia and growth faltering. Health centre clinicians reported these conditions to be commonly occurring and problematic to manage.

**Table 1:** Infant data collection and guidelines

**Anaemia diagnosis criteria**	**Haemoglobin (Hb) <110g/dl**	
**Anaemia monitoring**	Recorded Hb between 6–12 months	
	Hb at 1^st^ diagnosis of anaemia	
	Age and weight at 1^st^ diagnosis	
**Treatment guidelines for anaemia**	Dietary advice	
	Albendazole (parasite) treatment: (given for 3 days)	
	Iron treatment: (Type of treatment, number of intramuscular doses)	
	Folate for Hb <9gm/dl	
	Follow up haemoglobin after 4 weeks	
**Poor growth/ growth faltering criteria**	A child is not growing well if their plotted weight does not follow the shape and direction of the centile growth curves on the growth charts of if there has been no weight increase for:	
	**Age**	**Time of no weight increase**
	<2 months	2 weeks
	2-5 months	1 month
	6 months to <3 years	2 months
**Growth monitoring**	Record of ‘Road to Health’ chart	
	Record of Growth Action and Assessment (GAA) form (used for recording of Hb, weight, height and head circumference)	
	Number and timing of GAA visits	
	Weight, height and head circumference at each GAA visit	
**Treatment guidelines for growth faltering**	Where growth faltering identified, intervention recorded:	
	Additional growth monitoring	
	Nutritional education	
	Supplemental food for growth catch up	
	Medical checklist	
	Paediatric referral	
	District Medical Officer (DMO) referral	
	Growth Action Plan*	
	Community support services (e.g.: community/early childhood programs that support child health, feeding programs, referral to family support workers)	
	Vitamin A	
	Hospitalisation for failure to thrive	
	Family meeting	

*A Growth Action Plan was designed for implementation by the clinicians to ensure timely and appropriate interventions for the infant as soon as growth faltering was detected.

#### Interview data

The first author conducted 24 semi-structured interviews with clinicians who provided or managed child health services in the two remote study sites (Table 2). Seventy one percent of clinicians (*n =* 17) were resident in the remote community; the remainder based in the regional centre; providing outreach services. Initial purposive sampling recruited 17 clinicians with snowball sampling recruiting a further seven clinicians. Recruitment continued until data saturation had been achieved in the analysis. Interviews included core questions about the clinicians’ role, experience and views of remote infant health services and barriers to service delivery.

**Table 2:** Interview participants

**Place of employment**	**Clinicians (*n*** **= 24)**
HC 1 (*n* = 9)	District Medical Officers (*n* = 2)
	Remote Area Nurses (working in general roles) (*n* = 2)
	Remote Area Nurses (working in child health roles) (*n* = 2)
	Aboriginal Health Workers (*n* = 1)
	Managers (*n* = 2)
HC 2 (*n* = 10)	District Medical Officers (*n* = 2)
	Remote Area Nurses (working in general roles) (*n* = 2)
	Child Health Nurses (working in child health roles) (*n* = 3)
	Aboriginal Health Workers (*n* = 1)
	Managers (*n* = 2)
Regional Centre (*n* = 5)	Outreach (visiting) Child Health Nurses (*n* = 2)
	Outreach (visiting) Paediatricians (*n* = 3)

#### Data analysis

Medical record data were entered into an Access (™Microsoft Corporation) database, cleaned and analysed using Stata version 12.1 (™Statcorp, College Station, Texas). Continuous data are reported as means (1 standard deviation (SD), 95% Confidence Interval (CI)) or medians (Interquartile Range (IQR)). Dichotomous data are reported as proportions. Time to event data are presented using Kaplan-Meier estimators, and p-values derived using log-rank test. Z-scores based on World Health Organization (WHO) Child Growth Standards were derived using WHO published software for Stata [31].

Interviews were audio recorded with participant’s consent and transcribed verbatim. Field notes written during and following interviews described the setting, participant’s behaviours, body language and non-verbal communication. Pseudonyms were used for anonymity. The transcribed qualitative material was analysed by the first author using content analysis in ATLAS T.I 5.4 (™Scientific Software Development GmBH, Berlin, Germany). The transcriptions were examined to identify issues and themes in the data, assigning codes to units of meaning apparent in each paragraph or sentence. Data were then consolidated into higher-level categories and core themes identified. Frequencies evident within the core themes were then ascertained.

## Results

### Anaemia

Guidelines recommend Hb monitoring at six monthly intervals from six months of age [27] ; 85% (*n* = 338) of infants with an available health record (*n* = 398) had at least one recorded Hb between 6–12 months. Anaemia prevalence among all infants was 68% (*n* = 228) (95% CI 62.6-72.7); mean Hb 97.3gm/dl at first diagnosis (SD 9.3, 95% CI 96.1-98.5) when the mean age at diagnosis was 7.6 months (SD 2.8 months, 95% CI 7.3-8.0). The proportion of infants anaemic did not vary by prematurity status (Term 68.0%, pre-term 68.3%, p = 0.97).

Twenty percent of anaemic infants (n = 48) had documented dietary advice, 27% (*n* = 62) received a complete course of Albendazole supervised by a HC staff member. One third of infants (*n* = 68) received a completed course of iron (1–3 IM injections based on age and body weight) and 28% (*n* = 65) did not receive any iron treatment despite having documented anaemia. A follow-up Hb was checked in 60% of anaemic infants (*n* = 137). Less than one third (*n* = 11/42) of infants with an Hb <9gm/dl received folate.

### Growth faltering

Hard copy ‘Road to Health’ growth charts based on international references [32] were used for growth monitoring during the study period. Guidelines recommended regular growth monitoring and immediate intervention for faltering, commencing from birth. Growth faltering was documented by clinicians in RHC records of 42% (*n* = 167) of infants by age 1.

### Z-scores

**Figure 1:**
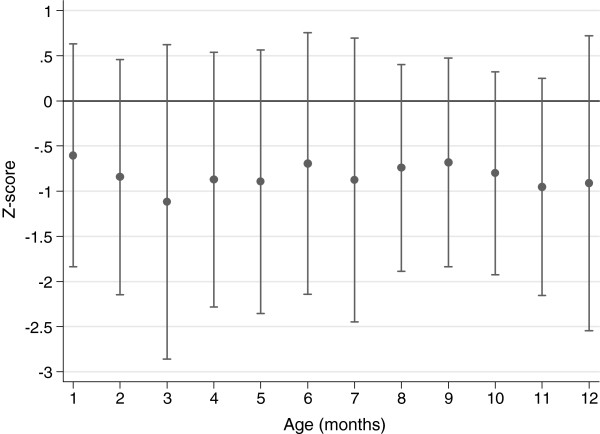
**Mean (SD) weight for age Z-score **≤ **−2 by month of age.**

**Figure 2:**
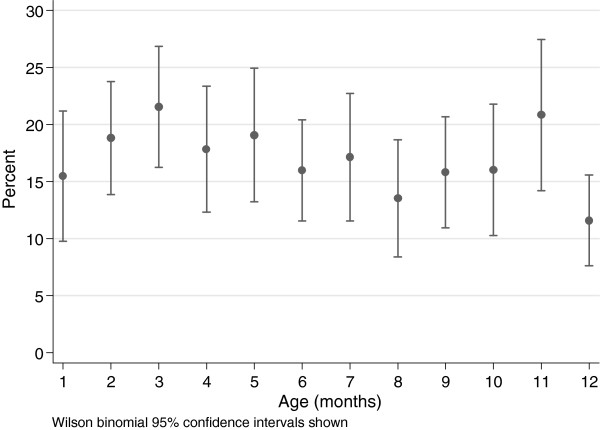
**Percent of infants with weight for age Z-score** ≤ **−2 at each month of age.**

**Figure 3:**
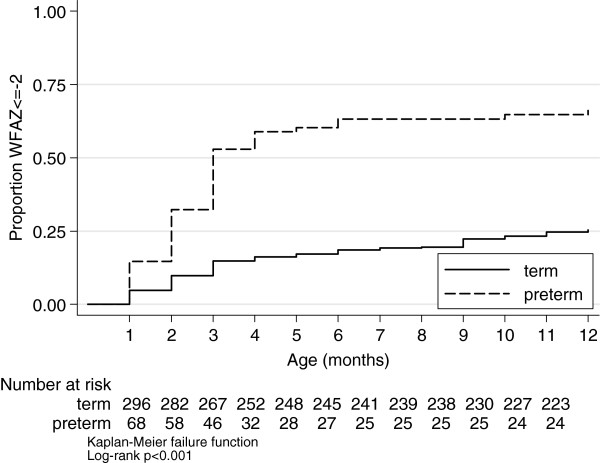
**Proportion of infants having weight for age Z-score ≤ −2 at least once.**

There were 2346 monthly observations of weight recorded for 372 infants, median number of observations per child was 5 (IQR 4 to 9). There was no evidence for a difference in mean visits by prematurity status (term: 6.1, preterm: 5.9, p = 0.62), or by underweight (>0 episodes weight for age Z-score ≤ −2: 6.1, no such episodes: 6.6, p = 0.12) . The mean weight for age Z-score by infant was −0.80 (SD 1.3) (Figure 1). There was no significant difference by gender. Marginal population mean (SD) prevalence of weight for age Z-score ≤ −2 at each monthly visit is shown in Figure 2. Among 372 infants there were 398 observations of weight for age Z-score≤ −2 of which 122 (122/372 = 32.8%) were first episodes, with mean age at first occasion being 3.9 (SD 3.1) months. Among 296 term infants, 75 (25.3%) had at least 1 episode weight for age Z score ≤ −2 among whom mean age at first episode was 4.4 (SD 3.3) months. Among 68 preterm infants, 45 (66.2%) had at least 1 episode among whom mean age at first episode was 2.9 (SD 2.2) months, p = 0.008 (Figure 3). For 940 observations of length among 354 infants, the mean length for age Z-score was −0.91 (SD 1.6) and for 931 observations of concurrent weight and length, the mean weight for length Z-score was −0.21 (SD 1.4).

Analysis of Z-score data revealed a high proportion of infants underweight, stunted or wasted in their first year (Table 3). Authors 1 and 2 independently analysed the change in monthly Z-scores for each of 374 infants and identified growth faltering (defined as any drop in Z-score) in 322 (86%) compared to 42% in whom faltering was documented by clinicians in RHC records. Of the 374 infants with two or more recorded weights: 55 (14.7%) had no growth faltering, 167 (44.6%) had a loss of less than 1 Z-score, 126 (33.6%) lost between 1 and 2 Z-scores, 24 (6.4%) lost between 2 and 3 Z-scores and 2 (0.5%) lost greater that 3 Z-scores.

**Table 3:** Underweight, stunting and wasting in first year of life

**Anthropometric measure**	**Infants**	**Proportion of cohort with overall mean Z-score **≤ **−2 for first year of life**	**Proportion of cohort with at least 1 episode of Z-score **≤ **−2 in first year of life**
Underweight (weight for age)	372	55 (14.8%)	122 (32.8%)
Stunted (length for age)	354	58 (16.4%)	97 (27.4%)
Wasted (weight for length)	354	19 (5.4%)	65 (18.3%)

Among all infants with growth faltering (*n* = 322), less than half received additional growth monitoring. Delivery of other interventions recommended in guidelines was often low (see Table 4) and the quality of documentation in the health record regarding the intervention and follow up management plan was often poor and incomplete.

**Table 4:** Proportion of infants with growth faltering identified by health worker who received an intervention

**Intervention**	**Received intervention**	**Growth faltering documented by clinician n = 162**	**Growth faltering identified by this study n = 322**
Extra growth monitoring	154	95%	48%
Nutritional advice	110	68%	34%
Nutritional supplements	65	40%	20%
Medical checklist	61	38%	19%
Referral to paediatrician	58	36%	18%
Referral to District Medical Officer	48	30%	15%
Growth Action Plan	48	30%	15%
Community support/services	24	15%	7%
Vitamin A/Zinc	21	13%	7%
Hospitalisation for failure to thrive	19	12%	6%
Family meeting	16	10%	5%

### Barriers to remote health service delivery

Interviews were undertaken to validate and explain our quantitative findings. Analysis of interview data revealed clinicians’ perspectives on barriers to health service delivery. These were particularly related to institutional factors and staff capacity. All clinicians interviewed recognised the quality of care for these infants was suboptimal.

### Organisational structure

Clinicians identified poor organisation and inadequate staffing of the RHCs as significant barriers to health care delivery. Each RHC had one or occasionally two nurses designated to provide primary child health care services to the under 5 population (approximately 300–320 children, of which 60–80 were infants). One RHC provided the primary health service on a part-time basis; the other provided a full time service.

Given the high volume of acute and complex presentations in both RHCs, additional HC staff were frequently required to assist with clinical management of infant cases. This interfered with the ability of staff to provide continuity of care and effective follow up of infants with identified problems. For example, an infant presenting to the RHC over a number of days could be seen by a different staff member at every presentation. Rarely did infants who presented with an acute illness receive routine or overdue health assessments unless designated child health clinicians saw them. Most non-designated child health clinicians did not view growth assessment, immunisations, anaemia checks or follow-up treatment as their responsibility, as one clinician observed:

‘..the child health nurse will write [in the medical notes] ‘ this child needs a Hb at the next check’ but the kid’s seen for acute presentations 20 times in between and it’s only when they get back to the well baby clinic (primary health service) that they get that Hb’ (F1)

The high turnover of staff was also perceived to compromise continuity of health care delivery. During the eight-month data collection period, each RHC had 5–7 different nursing staff rotating through the child health services; 75% of these were on short-term contracts (2 weeks - 6 months).

### Medical models of care

The high burden of disease in both communities was thought to have contributed to the health service’s long-standing focus on acute care and lesser value placed on preventative health care and education. This was clearly demonstrated among the mothers of these infants, whereby 31% of mothers were identified to be smokers at their first antenatal care visit yet only 8% received any smoking cessation advice [33]. This focus on acute care was also evident in the delivery of child health services.

One clinician stated: *‘We see the same kids week in week out with respiratory illness..We give them antis [antibiotics] and send them home. This is a major issue here. Yeah, I guess you know you are never going to fix these kids as we don’t deal with the real problem…the preventive stuff like everyone in the house smoking all the time..’ (S3)*

Linguistic and cultural barriers, including a lack of interpreters and culturally appropriate health education resources; and the complexity of family dynamics, were also noted to compromise effective health service delivery.

### Inadequate staff knowledge and skills

Only one nurse interviewed had formal child health qualifications, two had not previously worked with children and three were working in a remote health service for the first time. The lack of child health knowledge and skills specific to the needs of Aboriginal children particularly among nursing staff were compounded by reports of inadequate orientation to the health service and a lack of familiarity with the use of guidelines and surveillance tools, such as growth monitoring charts and limited opportunities for ongoing education or mentoring and supervision by senior staff.

More than half of the RHC based nurses interviewed did not feel competent to provide culturally appropriate health care to Aboriginal families and reported difficulties managing failure to thrive and nutritional issues. Illustrating this point, one participant noted:

‘I don’t know how to challenge families about feeding issues..you know the kid isn’t being fed properly but I feel like if you say this, it’s just shame (public embarrassment) and they’ll just think ‘ stuff her’ and not come back..’ (L5)

### Lack of Aboriginal staff

Many clinicians described the lack of AHWs, senior Aboriginal women or other local Aboriginal community workers involved in the delivery of infant health care services. In both RHCs, AHWs were predominantly working in administrative roles, despite their extensive clinical experience. Clinicians reported a steady decline in the number of AHWs and in their scope of practice. All non-Aboriginal clinicians described AHWs as imperative to effective health service delivery given their experience, relationships with local families, language, cultural and community knowledge.

Other barriers to service delivery, less frequently reported, related to the family’s responsibility to attend for care when required for follow-up treatment. Families often spent time away from their home communities; looking after family members in hospital in Darwin, attending funerals, ceremony or bush holidays and did not present with their infants for care when this had been planned. Traditional Aboriginal cultural ceremonies were significant in both remote communities and mourning and funeral obligations taken seriously. These often involved families relocating to stay with family in mourning or where the ceremony was being held for extended periods of time, sometimes for up to several months.

Families were usually dependent on transport provided by the RHC to attend follow-up appointments, as there was no public transport available. At times, a lack of drivers or vehicles meant that families were not brought to the HC when required. On other occasions, drivers or clinicians themselves would present numerous times to the families home but they would either not be there or refuse to be transported to the HC because the timing was inconvenient. Clinicians also reported visiting families who were known to be ‘poor attendees’ at their home to ‘encourage’ their attendance. These strategies fail to increase the capacity of families to take responsibility for the health of their children though clinicians often reported the dilemma of not ‘chasing up families’ versus the rights of the child to receive health care.

## Discussion

Access to quality primary health care services is a determinant of good health [34]. The findings from this study show remote NT health services are not providing effective management of anaemia nor adequate identification of growth faltering for Aboriginal infants.

Growth faltering and anaemia prevalence was higher than previously documented [20,21,35]. Growth faltering was documented by clinicians in 42% of infants, however by a definition of any reduction in Z-score over time we identified over twice that number. Whether seen as point prevalence at each monthly visit, or when examined as an overall mean low Z-score over all visits per child we detected a higher proportion of infants underweight and stunted than the Northern Territory GAA survey during the same period (12% and 7% respectively) [35], and the proportion of children with at least one episode was higher still. The NT GAA cross-sectional survey reports measurements per child at a single timepoint, whereas we followed the measurement of the cohort over 12 months. The fact that in our cohort the proportion with any episode of underweight is higher than the the proportion with overall mean underweight over 12 months may be accounted for in part by infants who falter several months into their first year and in part may suggest that some infants who become malnourished may improve. However, overall prevalence changes very little by month.

The rate of first episode underweight in the cohort was 32.8%, and growth faltering was found in 86% of infants. The number of observation visits did not vary by prematurity or underweight, but only only a small proportion of children had 12 GAA monthly visits. Excluding missed visits from person time observed gives an incidence of 820 per 1000 infant years, but including missed visits in observation (and assuming no underweight episodes in the interval censored observations) gives an incidence of 421 per 1000 infant years. We have shown that among Aboriginal infants in the Top End malnutrition is common, occurs early and is persistent over the first year of life.

Anaemia was identified in 68% percent of infants but only one third received a completed course of treatment for this. Another third of all anaemic infants failed to receive any treatment despite having this condition documented in the health record. Anaemia associated with pre-term birth is a common problem worldwide [36]. This has substantial clinical implications including the interference with normal growth leading to subsequent growth faltering [36] and recovery processes for respiratory diseases and bacterial infections [37], all highly prevalent problems in remote Aboriginal infants in Australia’s Top End [38]. However, in this study we demonstrated that anaemia was not associated with prematurity. Although we found a significantly higher and earlier incidence of WFAZ < = −2 among pre-term infants it should be borne in mind appropriate gestational age correction may not have been routinely done by health workers.

Poor identification and management of infant health problems was contributed to by staff not receiving adequate education, supervision, orientation to remote health services, lack of familiarity with local guidelines and inadequate skills in accurately or systematically plotting and interpreting growth. Electronic systems are now being used in many RHCs, where computer programs plot infant’s growth against international standards. This will lessen the risk human error in the plotting of measurements.

Remote dwelling Aboriginal infants access RHCs frequently from an early age [18,19] and clinicians were overburdened by the volume and complexity of presentations. This compromised their ability to provide good care. Poor organisation and utilisation of existing staff was identified, such the AHWs in these settings being used in administrative roles despite their experience and knowledge.

Lack of continuity, which in this study arose from high staff turnover, staff being moved between different health program areas and multiple (or absent) handovers of care, will increase errors and jeopardise safety [39]. Continuity of carer is a critical component of primary health care known to improve the quality of service delivery [40]. Studies indicate that continuity of carer at the primary care level reduces hospital admissions, improves compliance with treatment, increases preventive care and improves relationships between clinician and patient [41,42]. Maintaining skilled and knowledgeable continuity of carer can be challenging in this context given the high staff turnover and difficulties retaining staff in remote communities [43] but should be a priority.

Poor follow-up of infants with identified health problem in other remote health services across Australia has also been shown by Baillie et al. [2008] [20] reflecting the inability of current health systems to adequately provide for these Aboriginal populations. Low rates of adherence to local guidelines for the delivery of antenatal care and follow up of highly prevalent problems such as sexually transmitted infections, smoking and anaemia were also found in these two remote communities and the barriers to providing care similar to those described in this study [33].

There are a number of strategies that could help to improve the quality of care in these remote health services (see Table 5) such as service redesign that includes appropriate staffing based on service utilisation patterns and actual workload [44,45] with community based health service delivery by community workers (CWs) [46]. This might also reduce the high work load in HCs, allowing HC based clinicians to concentrate on providing acute care.

**Table 5:** Key strategies for improving quality of remote infant health care

	
**Service organization and delivery**	• Implement a culturally appropriate model of service delivery based on community development principals and continuity of care.
	• Provide flexibility in service delivery: times/location: home visiting, community based care
	• Increase delivery of community based health care interventions.
**Workforce**	• Staffing for health services based on patterns of service use, workload, and community health care needs
	• Scale up of designated child health nurses and community based family support workers.
	• Ensure effective integration and increase leadership of AHW staff in the health service.
**Education and training**	• Mandatory cultural security training undertaken by all clinicians prior to commencement of employment in remote communities. Inclusion of a component on Aboriginal child rearing practices.
	• Introduction of a minimum set of core competencies in child health for all clinicians that are assessed on an annual basis.
	• Ensure clinicians working with children are appropriately qualified to do so or be working towards obtaining a child health qualification.
	• Provide clinicians with opportunities to undertake distance education modules to build skills and knowledge directly relevant to remote area practice.
	• Ensure all clinicians have access to designated ‘specialist’ mentors or preceptors within and external to their workplace that can provide mentoring and opportunities for knowledge and skills refresher training in the workplace.
	• Ensure Aboriginal Health Workers and other community workers have a larger role in health education and health promotion activities or community-based interventions, such as for growth faltering.
**Clinical governance and leadership**	• Management to ensure all new and existing clinicians are orientated to the health service and trained in the use of the local guidelines, primary care manuals, referral practices and documentation. Ensure regular refresher training on use of guidelines and patient information systems.
	• Regular supervision of health care practice and auditing of documentation.
	• Establish key targets for health outcomes and service delivery performance specific to the needs of individual health facilities.
	• Implementation of local systems for regular monitoring and evaluation of child health outcomes and health system performance with action plan for facilitating improvements.

Evidence supports the need for early intervention in the treatment of growth and nutritional conditions. Community-based interventions however, involving carers and other CWs are effective in addressing underlying issues and prevent repeated episodes [47,48] and need to be considered as part of health service planning for this population. The use of carefully chosen, appropriately trained and well-supported CWs for the delivery of health education, basic primary health care and to provide linkages to health services has been a successful strategy in many under resourced settings [46]. In this study, we identified very poor engagement of clinicians with carers to address growth and nutritional issues and absence of community based services.

There is an urgent need for increased child health skills and knowledge with most nurses lacking qualifications or experience, despite working with a population who have among the poorest child health outcomes in Australia. In other Australian settings, unqualified staff would not provide this care. Over the past decade, there have been increased educational opportunities for RNs working in remote settings [49]. However, only 5% of the nurses who work in very remote Australian health services have specific skills and qualifications for their advanced practice role [29]. A lack of financial support to undertake further education, high workloads and on call hours makes continuing education difficult in this setting.

Cultural competency in health delivery can improve outcomes for Aboriginal mothers and infants [50,51] but attention to this was also absent. Further, understanding of child rearing practices also need to be available so any differences in parenting behaviours and values are incorporated into health messages.

Much of our data collection occurred during the NT National Emergency Response (also referred to as NT Intervention). This was a legislative response introduced by Australia’s federal government to tackle reports of sexual abuse and neglect of children in Aboriginal communities across the NT which saw changes made to the provision of welfare benefits, law enforcement, land tenure and restrictions on alcohol use [52]. A roll out of child health checks and follow up of primary health and specialist services were also introduced into remote communities. However as our data collection period ceased at the end of the 1^st^ year of life for infants born 2004–2006, few of the infants in our study were part of these checks. Further government funding was also provided to remote communities to expand primary health service delivery [53]. A number of new approaches are now underway in remote NT communities to improve child health services and improve quality of care including a new evidence based health care delivery program targeting under 5s (Healthy Kids Under 5 Program), designated qualified Child and Family Health Nurses who will provide community based care thus increasing flexibility and access to services and increasing the numbers of community-based family support workers. Also a newly developed and Indigenous focused Graduate Diploma in Child and Family Health is now offered by the local university, by distance learning. The quality of infant health care in our study sites following the NT intervention has recently been evaluated by the 1 + 1 study team with the results expected for publication in the coming year.

Following our data collection, many RHCs implemented continuous quality improvement strategies to strengthen primary health care services. These quality improvement strategies included monitoring of health performance and outcome indicators and providing feedback to clinicians to improve health care accountability [54]. The measures used here to assess to quality of infant health service delivery were developed as part of a broader set of indicators specifically for remote dwelling Aboriginal maternal and infant populations [55,56]. Regular montioring and evaluation using such indicators can serve as a useful way of RHCs assessing health outcomes, their own service delivery performance and taking accountability for system performance. The findings of this study have been reported to Local Reference Groups in the communities and to senior policy and clinicians in the NT as part of the participatory action research study design and have contributed to health system improvements.

### Limitations

In the NT, Aboriginal children and their families are highly mobile [57] and it is possible that the infants may have presented for additional healthcare at other RHCs not reviewed as part of this study and this may bias our results. Preventive health care is often opportunistic in remote health centres and some infants may have had their haemoglobin checked by a clinician if they presented at 5 months and anaemia treatment commenced at that visit. Our data collection did not capture these infants who may have had this care at an earlier date.

As our growth data were retrospectively collected from routine GAA visits forms as part of the remote health records we were unable to reliably determine whether age was corrected for gestational age among preterm infants. We suspect that correction was not always made, since the GAA program encourages health centre visits at each month of life following birth, and this may falsely inflate the rate of underweight. More non-GAA visits are often scheduled for preterm infants, but number of GAA visits did not differ by prematurity.

The protective effect of breastfeeding against infection [58,59] and growth faltering [60] is well founded. In this study we were unable to collect reliable breastfeeding data using the remote health centre records to examine for associations between growth faltering and breastfeeding. Breastfeeding status was not routinely documented in health centre records as part of health assessments nor was it recorded on the GAA form. Limited breastfeeding data was obtained from hospital discharge records where we found 88% of infants’ breastfed on discharge. This is comparable with other data for Indigenous and non-Indigenous infants across Australia during the same time period [61]. As part of the Healthy Under 5 Kids Program that has been implemented since the data collection in this study, information on breastfeeding is now recorded on every structured health assessment used from birth to age two [62].

## Conclusions

Australian Aboriginal infants have worse health outcomes than non-Indigenous infants and care provided for anaemia and growth faltering is of inadequate standard. These conditions are preventable, occur too frequently and are poorly treated. Service design, lack of continuity of carer and staffing organisation and capacity are contributing factors. These must be urgently addressed to reduce the unacceptably high disparities in health outcomes for Aboriginal infants.

## Competing interests

The authors declare that they have no competing interests.

## Authors’ contributions

SBZ was responsible for the study design, obtaining ethical approval, data collection, and data analysis and drafting the manuscript. NBZ assisted with data cleaning and analysis. SGK, participated in designing the study and assisted with data analysis. LB and SVK provided comments on the study design, analysis and the manuscript. All authors read and approved the final manuscript.

## Pre-publication history

The pre-publication history for this paper can be accessed here:

http://www.biomedcentral.com/1472-6963/13/250/prepub
